# “The Culturally and Linguistically Diverse Community Is Not Just Minoritised But Ignored”: Engaging Culturally and Linguistically Diverse Communities in Australia With Blood‐Borne Viruses and Sexually Transmissible Infections Healthcare

**DOI:** 10.1111/hex.70416

**Published:** 2025-09-04

**Authors:** S. R. Okeke, R. Horwitz, L. Brener, H. M. K. Vu, E. Wu, D. Jin, S. Yu, T. Broady, C. Treloar, E. Cama

**Affiliations:** ^1^ Centre for Social Research in Health UNSW Sydney Sydney New South Wales Australia

**Keywords:** cross‐cultural approach, cultural partnerships, health literacy, patient engagement, stigma

## Abstract

**Background:**

People from culturally and linguistically diverse (CALD) backgrounds who are living in high‐income countries, are disproportionately impacted by blood‐borne viruses and sexually transmissible infections (BBVs/STIs). Despite this, many do not engage with available preventive and treatment services due to a range of patient, provider and systems level barriers that make patient engagement challenging. This study explores ways to make healthcare services more acceptable and accessible to promote better health outcomes for Australian residents from CALD communities.

**Methods:**

Researchers of CALD backgrounds conducted in‐depth interviews with 26 key stakeholders who work in the BBVs/STIs health care sector and advocate for or provide care to people from CALD communities. Most of these stakeholders were of CALD backgrounds. Interviews explored barriers to patient engagement, and discussed notions of intersectional stigma, quality health care, and strategies to reduce stigma and discrimination within health settings. Data were analysed using reflexive thematic analysis.

**Results:**

Four key themes were generated that describe and challenge inequitable BBVs and STIs care for CALD people in Australia and offer more inclusive and acceptable paths of engagement for this population. These themes are: (1) A paradigm shift in cultural orientation of the Australian healthcare system, (2) Addressing stigma, (3) Removing language barriers, and (4) Engaging CALD people as healthcare providers.

**Conclusion:**

Results show that health service understanding of CALD people's cultural understandings and interpretations of health and illness is key to engaging these communities with healthcare services. This study highlights the need for BBVs/STIs services and Australia's health system in general, to adopt a more cross‐cultural approach in the way it interacts with and addresses the health needs of patients from CALD backgrounds.

**Patient or Public Contribution:**

This study used a codesign approach in which an advisory group drawn from CALD communities made a significant input in the study design in relation to cultural appropriateness and relevance. Also, some participants in the study have BBVs living experience which contributed in enriching the study data.

**Abbreviations**
AIDS: acquired immunodeficiency syndrome
BBVs: blood‐borne viruses
CALD: culturally and linguistically diverse
GP: general practitioner (a medical doctor providing general services)
HBV: Hepatitis B virus
HCV: Hepatitis C virus
HIV: human immunodeficiency virus
LGBTQIA+: lesbian, gay, bisexual, transgender, queer, intersex, and asexual, plus
RTA: reflexive thematic analysis
STIs: sexually transmissible infections

## 1 Background

Due to the controversies surrounding the label, “culturally and linguistically diverse (CALD)” [1, 2, 3], we begin with a contextualisation of the term in relation to how we used it in this study. Historically, the term CALD is traceable to efforts in the late 1990s to distinguish Australian residents who are neither indigenous nor of the Anglo‐Saxon ancestry [1, 2]. The use of this nomenclature in public and population health presents both opportunities—for more representation and equitable research and intervention efforts—as well as challenges of stereotypical and deficit‐based connotation. A greater focus on the challenges of the CALD term has resulted in justifiable criticisms and contestations [1, 2, 3]. Interestingly, a critical analysis of the issues raised in these contestations indicates that the contention is more on how the term is being used than in the term itself. As such, when the term is used in a more critical and strength‐based manner, it could help “address diversity,…guide equitable access to health resources, and inform inclusive policies and programs” [1]. It is against this backdrop that we have adopted and used the term in our research.

Our approach to using CALD does not however ignore or dismiss concerns relating to how the term may homogenise a range of different perspectives and experiences [1]. We affirm that people from CALD communities in Australia may hold diverse and varying views on health and illness and that these differences may be a function of a range of issues ranging from migration histories to country of birth, education level and even social class. While there may be controversies surrounding CALD as a term [1, 2, 3], there seems to be consensus that people from CALD communities in Australia [4, 5, 6, 7, 8], just like other ethnic minorities and migrant groups in high income countries [9, 10, 11], are disproportionately impacted by adverse health outcomes.

Despite this disparity, there is comparatively low access to preventive and therapeutic health care services among members of these communities [12, 13, 14, 15]. Researchers have documented numerous barriers that influence access to health care among CALD communities in Australia. Some of these are patient‐ and provider‐level barriers such as poor health literacy, multimorbidity (the presence of two or more chronic conditions in a person at the same time), and language and communication barriers [12, 13, 14]. There are also system‐level barriers, such as the complexities of navigating the health system and the inability to provide culturally appropriate services, which further constrain healthcare access among these communities [16, 17, 18]. In the Australian context, there is extensive evidence indicating that CALD people's unfamiliarity with and stress associated with navigating the complex health care system constitute a major barrier to patient engagement and health care access among these communities [12, 13, 19, 20, 21, 22].

Patient engagement has been defined as both desire and capability to actively participate in health care in a way appropriate to the individual, in cooperation with a healthcare provider or institution, for the purposes of maximising outcomes or improving experiences of care [23]. Patient engagement has been included in healthcare improvement models as a strategy to address barriers to health care access and improve quality of care and patient outcomes [23, 24, 25, 26, 27]. Numerous studies have shown that patient engagement improves treatment safety and quality, as well as patients' adherence to treatment regimens, treatment outcomes, and patients’ satisfaction [28, 29, 30].

Patient engagement can be facilitated or constrained at patient‐, clinician/provider‐, and health system‐levels. At the individual level, engagement may be constrained by low health literacy [31, 32, 33], although it is important to stress the role that providers play in effectively communicating with patients, including those with low health literacy [34, 35]. At the provider level, patient engagement may be constrained by power imbalances between provider and patient (largely due to medical paternalism) [36, 37] and finally at the system level, patient engagement may be hindered by structural barriers co‐existing with and/or compounded by racial discrimination alienating or minoritising patients from CALD communities [38, 39, 40, 41]. Also, at the health system level and especially in developed countries, people from CALD backgrounds may experience unique barriers to patient engagement. Socio‐cultural differences between CALD and mainstream populations in understanding health and illness [39, 40, 41] make patient engagement even more difficult for CALD people [13, 40, 42]. It is therefore important to understand how to reduce barriers and strengthen facilitators of patient engagement especially for CALD communities in Australia, who are known to be experiencing multiple and intersecting vulnerabilities [13, 27]. One strategy to realising this goal is for the Australian health system to adopt a cross‐cultural approach which reflects the cultural diversity of the Australian society [40]. A cross‐cultural health system approach aims to reconcile different health‐related cultural values, behaviours, and practices to accomplish an inclusive and effective healthcare across different cultures [40, 43].

Making preventive and treatment services for blood borne viruses (BBVs) and sexually transmissible infections (STIs) accessible for people of CALD backgrounds in Australia is important because this population is known to be disproportionately impacted by these conditions [5, 44, 45, 46] and are listed as a priority population in the national strategies for hepatitis B [47], hepatitis C, HIV [48], and the national BBVs and STIs research strategy [49]. For instance, people from CALD backgrounds account for 70% of Australian hepatitis B cases [5]. Also, when compared with people born in Australia, there is a higher HIV notification rates among people born overseas [44]. A large body of evidence shows BBVs and STIs are highly stigmatised conditions among people of CALD backgrounds in Australia and other high‐income settings, especially among those from contexts where conservative sexual and socio‐cultural norms are predominant [14, 42, 50, 51, 52, 53, 54, 55, 56, 57]. Anticipated and/or actual BBVs and STIs stigma can uniquely impact the well‐being of CALD communities members as prevalent traditional socio‐cultural and religious beliefs may deter engagement with BBVs/STIs preventive and treatment services [57, 58, 59, 60].

Identifying the importance of collectivism to one's identity––in which bonds to family, community, and culture prevail and where the actions of an individual are often intricately linked to their family and broader community––is important in understanding experiences of illness and healthcare [61, 62]. Moreover, in addition to actual or anticipated stigma at the community and the health system levels, people of CALD backgrounds may also experience other forms of stigma and discrimination such as stigma associated with: migration status (racism) [11, 15, 57], sexual orientation [62, 63], gender [64, 65] and a combination of two or more of these and other factors, resulting in intersectional stigma which could further compound low patient engagement among this population.

Notably, health services in Western societies, in many cases, fail to consider the complex intersecting and unique factors constraining CALD people's engagement with health services [15, 66, 67, 68]. By failing to consider the complexities surrounding attitudes towards and engagement with BBVs and STIs treatment and care, healthcare services in high‐income countries seem to minoritise CALD communities [11, 15, 69, 70] as patient engagement for members of these communities become further constrained at the health system level. Given the many barriers that CALD communities face in health care [11, 13, 15, 67, 69, 70], understanding how to engage these communities with preventive and treatment services is needed. This study therefore aims at gaining greater insights from key stakeholders who advocate for and work with CALD communities to inform how to design, implement, and evaluate accessible, acceptable and sustainable BBVs and STIs preventive and treatment services for these communities in Australia.

## 2 Methods

### 2.1 Design and Paradigm

This qualitative research–reported using relevant aspects of the Big Q Qualitative Reporting Guidelines [71] –is guided by a combination of interpretivist and critical research paradigms. Thus, participants' perspectives and shared experiences are not only subjectively constructed, but they also reflect participants' and researchers' dialogic reflections around how to challenge inequity, racism, and exclusion within the Australian healthcare systems [72, 73].

To support the aims of the research, all of the interviewers and most of the participants are members of CALD communities in Australia, and as such, assume insider‐outsider positionality. This positionality confers experiential knowledge and deep understanding of CALD people's experience, particularly those with lived/living experience of BBVs. The insider‐outsider positionality of members of the research team and most participants reflexively shaped the overall study and knowledge claims [74] and strengthen the research methodology [75].

### 2.2 Study Setting and Sample

This qualitative study generated data through in‐depth semi‐structured interviews involving 26 key stakeholders who work with, advocate for, or provide care to people from CALD communities. These participants were also largely from CALD backgrounds themselves, although this was not a prerequisite for taking part in the research. The interviews were conducted between June and December 2023. Researchers identified key participants/stakeholders based on their professional networks and through snowballing. Stakeholders included people with previous or current clinical experience in BBVs and STIs care, cultural workers from community organisations related to BBVs and STIs, people working within organisations that support migrants and refugees, healthcare professionals and academics with expertise in the BBVs/STIs and health service research spaces. Approval for the study was granted by the University of New South Wales Human Research Ethics Committee. All participants provided informed consent before interview through either signing the consent form or providing verbal consent before the interview commenced. Participants were reimbursed with $50 gift voucher to compensate for their time.

### 2.3 Data Collection

The interviews were guided by a semi‐structured interview guide. Five researchers, all from different CALD backgrounds, conducted the interviews in English via telephone or Zoom. The interviews explored perceptions of stigma, what quality health care should look like, and how to reduce stigma against CALD communities within health settings, including practical considerations for improving patient engagement in BBVs/STIs services among people from CALD communities in Australia. All the interviews (lasting between 60 and 90 minutes) were audio‐recorded and transcribed by a professional transcriber operating under a confidentiality agreement. Transcripts were then checked for accuracy by the interviewers, with any identifying information removed from the transcripts.

### 2.4 Data Analysis

Data were analysed using reflexive thematic analysis [74, 76]. Since this is an exploratory study, analysis of the transcripts for theme generation involved inductive, data‐driven, and bottom‐up approaches [77]. Consequently, themes represent patterns of shared meaning [75], instead of domain summaries from the interview guide [78]. Analysis began with multiple reflexive and reflective reading of the data transcripts and field notes for immersion and familiarisation beyond semantic connotations. Transcripts were then coded using the NVivo software. Four key themes were generated from the transcribed data to represent key strategies participants believe could improve engagement with BBVs and STIs services among CALD communities in Australia. These themes are: a paradigm shift in cultural orientation of the Australian healthcare system; addressing stigma; removing language barriers; and engaging CALD people as healthcare providers.

## 3 Results

### 3.1 Sample Characteristics

The sample consisted of 26 participants, of which 17 identified as women, seven identified as men, and one participant identified as nonbinary. Eleven participants were recruited from NSW, five were recruited from Victoria, four from South Australia, three from Western Australia, two from Queensland and one from Australian Capital Territory. Four of the participants reported lived/living experience of BBVs/STIs.

### 3.2 A Paradigm Shift in Cultural Orientation of the Australian Healthcare System

Participants were unanimous that to increase patient engagement among CALD people attending services for BBVs and STIs, there would need to be a shift in the current cultural orientation of the Australian health service. There was a clear consensus among participants regarding a clear difference in worldviews of people of CALD backgrounds when compared to the Western worldviews that is dominant in Australia. This difference, as shared by participants, has made engaging with healthcare in Australia challenging for people of CALD backgrounds.

Participants reported that they felt there were differences between the way the Australian health care system function with a focus on individual responsibility for health, compared to collective culture that is more common among many CALDcommunities. Further, the Western notion of individual responsibility for health, places responsibility on the individual to seek help for themselves when they are ill. Participants' perspectives suggest that this challenge is foundational to the difficulty people of CALD backgrounds experience in navigating healthcare in Australia:

…the basic understanding is that we are trying to deal with people who come from collective cultures with an individualistic approach… The problem is that the [healthcare] system relies on individuals to come and seek help. So, there is an assumption that a person knows their needs and that the person will seek help when they are in need

(P2/female)

Though diverse and heterogeneous, CALD communities share, to a large extent, a collectivist cultural orientation [60, 79, 80]. This orientation somewhat emphasises strong communal ties with implications for not disclosing positive BBVs and STIs status for fear of stigma‐induced social isolation [60, 81]. This fear could also lead to delayed testing, serve as a barrier to engaging with treatment and accessing social support [60]. However, this collectivist orientation, when properly harnessed, could also prove useful in improving these communities' engagement with health services. For instance, one participant shared an anecdote of how this collectivist approach was strategically used to engage a group of people from CALD communities to access BBVs and STIs health services.

So, [there is] an example of a group of same‐sex or men who sleep with men, who are from multicultural background. We understood they are not going to go individually to the sexual health clinic to have their regular testing for blood‐borne virus, but they will go if they were a group of them. So, we organise[d] trips. We organise[d] trips [with] our peer worker. [We said] “We'll meet you in botanic garden and we'll have a meal together and then we all go to sexual health clinic.” So, those are major differences, in how we approach a situation

(P3/female)

Identifying the importance of collectivism, where the actions of an individual are often intricately linked to the broader community, is an important step in increasing access to and engagement with care in any CALD communities where collectivism is predominant. Collectivist cultural orientation places emphasis on community action for communal interest. When public health messaging and healthcare services are designed to appeal to community action and interest, people of CALD backgrounds may be more likely to engage. One participant used the COVID‐19 experience to support this point:

So, if you say you're doing this and this to protect you and the community, they're like, yeah, I'll do it. I'll definitely do it because whatever you do to enhance the better opportunity for your family, for your community, most people will want to go and do that… It's about enticing them to do the right thing for the community, my community. Then they say yes, because you remember during the COVID time, a lot of people stayed home because they didn't want to infect other people. They wanted to protect the elderly people, immunocompromised people from getting COVID. That's why they stayed home. They wear masks because they didn't want to infect people. So, when you appeal to people about the sense of community responsibility, it makes a huge difference in their incentive of how to do and how to look after their health”

(P20/female)

Similarly, to create better engagement, using a collectivist approach such as storytelling or other more culturally significant means of disseminating health information could be more productive for any CALD communities that value the collectivist approach over the Eurocentric individualistic approach, that is, more common in Australia. This perspective was vividly shared at the interviews:

…because multicultural [people]… come from collectivist society, which means that we learn more through story‐telling, we learn more through group, through discussion, not necessarily through reading

(P2/female)

Moreover, using the less‐formalised and collectivist‐based approach of community, instead of the structured and formalised individualistic‐based approach could also improve some CALD communities members' use of health services.

If we could provide healthcare in more of a community setting, not so formal, then we go and like we're doing ourselves, we go to the community, we meet in a library, we explain health topics. If doctors could approach the CALD community more in a more casual way, we may be more successful because in our communities, let's say we are not so formal. To make it formal, I need to do all these forms and I need to call you to make an appointment. Try to offer more casual approach to health, then the person may feel comfortable to share with you what's happening

(P3/female)

Participants also shared experiences and advocated for representation of people of diverse backgrounds in healthcare settings and in health promotion materials to make the health system more inclusive by making the service more welcoming and less threatening as one participant observed:

Well, maybe things with the language of people like posters…just things around that are in culturally…like different cultures have different languages, different pictures, different…just, yeah not always just the white Australian, just multicultural information and when you look around, you know when you look around the room, it's not just white faces

(P16/female)

In addition, the complexity of the Australian health system can be overwhelming for people from CALD backgrounds, especially for newly arrived migrants or refugees. The multi‐layered nature of the system—comprising private and public services, various referral pathways, and a range of healthcare professionals—can be complex and challenging to navigate. To address these barriers, services must work towards helping CALD people to navigate these complex systems.

I think it's very difficult because the health system is so complicated. I think probably even the local people have trouble sometimes, let alone people from another country and also some people actually are migrants and have different health system compared to Australia. For example, like China, you know, we don't have a GP there. If people get sick, they just go to the hospital straightaway, but here you have GPs. You have to go to GP first, for example. That's just one example of very different health systems and also very complicated, like specialists and general practitioners, nurse practitioners, psychologists, you know, allied health providers as well. So, yeah, it's very complicated and add to that, you know, the language barrier and, you know, all these barriers, it's more difficult for them to navigate the health systems

(P24/female).

Furthermore, structural issues such as the lack of time and adequate resources in healthcare settings can exacerbate these issues, making individuals from CALD communities to feel neglected.

…it's time‐consuming sometimes when people…the communication is difficult, it takes time and understanding, and that's when it can fall down because staff are in a hurry or they're understaffed, or they just haven't got time to deal with that…sometimes they feel like they're not being attended to, ignored, overlooked, not understood. It's too hard

(P16/female).

Participants also stressed the importance of addressing power imbalances between health service providers and patients. In particular, health providers' expectations of patients' English language proficiency can make CALD communities members see the health service as unsafe and intimidating to navigate. When power imbalances exist, patients may feel disempowered, unheard, or reluctant to ask questions or express concerns, all of which contribute to poorer health outcomes and lower patient engagement and satisfaction.

There's always that hierarchy in the healthcare where the doctor knows best and they will be the one that prescribe things to you, and you are a patient here. You listen to him. This power dynamics is very intricate. I mean it's really hard to dismantle as well…you feel like, “Oh, I feel bad if I keep asking questions. The doctor will feel offended if I keep asking questions” or the doctor says that or asks you back, “Are you a doctor? Are you a trained medical professional? Why are you asking me all these questions? You listen to me. This is my prescription for you. This is what you have to do” and things like that. They have that general idea, so they don't question things. Well, when the doctor says so, that's the Bible… There will be a lot of balancing needed and training needed not only for the healthcare professional, I think, but also for the people in general to know that they do have a right and they do have a say in their health needs, and the power dynamics should be shifted back to the balance

(P21/male/BBV living experience)

Stigma experience occurring at both community and health system levels may further reinforce a feeling of alienation and poor engagement with BBVs/STIs healthcare services for patients from CALD communities as explored in the next theme.

### 3.3 Addressing Stigma

BBVs and STIs are highly stigmatised conditions. People of CALD backgrounds may experience intersectional stigma, including towards race, gender, or sexual orientation, thereby making stigma experiences multifaceted for people from these communities and acting as a barrier to health care access [14, 54].

As may be common with other stereotypes in the larger Australian society, BBVs/STIs within CALD communities, are usually attributed to a failure to uphold traditional socio‐sexual norms [57, 82]. This attribution precipitates and reinforces stigma against community members living with these conditions. This attribution could also discourage members of these communities from seeking BBVs/STIs‐related testing, treatment, and care, as they may fear judgement or rejection from their communities. This, in turn, could perpetuate cycles of poor health outcomes and may even lead to members of these communities avoiding disclosure of a positive diagnosis to others, including healthcare providers. One of the participants clearly shares how the fear of stigma and discrimination can impact access to and engagement with BBVs/STIs services among members of CALD communities in Australia:

HIV is very unique in this way because when we work with people in particular from non‐Australian backgrounds…very little has changed in these communities in terms of awareness and acceptance of HIV… So, what we're seeing is that those issues we would expect people to have sort of a higher knowledge potentially of transmission and risk is not changing, and it's not changing because people are too frightened of what is going to happen if they disclose their diagnosis… they're frightened of social ostracization. They're frightened of being excluded… and therefore it almost stops any dialogue at all, any education or anything from entering into those communities that might actually, bring about some change of understanding and awareness. In other areas, in terms of health, there is a lot of understanding and health literacy around other issues, but when it comes to these very stigmatising illnesses and chronic conditions, it feels as if time has stopped

(P7/female)

The comment above clearly demonstrates how attitudes towards BBVs and STIs within some CALD communities may still be conservative and highlights the need for anti‐stigma messaging designed specifically for such CALD communities to address these notions so that people feel comfortable to discuss and disclose their BBVs/STIs status and history without fear of social exclusion. A participant described this entrenched stigma and discrimination as a “golden curtain” that must be dismantled, if people of CALD backgrounds are to meaningfully engage with and utilise healthcare. The imagery of “golden curtain” of BBVs also involves assumptions and judgmental attitudes against people living with BBVs/STIs:

If there is still a stigma, if there's still like the pictures of AIDS or hepatitis or anything like in the ’90 s, you won't be able to achieve anything. So, I think first thing first is that we need to dismantle the Golden Curtain around all these BBV things… so dismantling the old perceptions of “HIV equals that, hepatitis equals that, STI is because you're promiscuous, you're naughty, you are not following the word of the society”

(P21/male/BBV living experience)

Perspectives shared during the interviews indicate that addressing stigma also involves challenging the socio‐cultural and religious norms and assumptions that drive stigma and discrimination. The process of addressing stigma is not about trying to change cultural or religious values, but about ensuring that those values do not become a barrier to care or an excuse for people, including healthcare providers to become judgmental of other people choices and practices. By respecting and navigating cultural boundaries with sensitivity, healthcare providers can help create a more inclusive and supportive environment for all patients.

…there's nothing wrong being religious, but if you are religious, then you have certain norms of society fairly or things embedded within you. There's nothing wrong with that. The issues start when you start imposing that belief to other people…if every single GP, every single healthcare professional, every single human being actually knows [to] keep whatever is within you, within you. You work within the boundary. So, the healthcare professionals should be really trained to understand that people are different, everybody is different. Not everyone is going to follow your norms, your expectations and things like that

(P21/male/BBV living experience)

The interview data also showed the complexity of seeking healthcare from providers of CALD backgrounds and highlights how healthcare providers' own cultural and religious values can shape the way they interact with patients. While it's commonly assumed that people from the same cultural or ethnic background will be more empathetic and understanding towards others in their community, providers' personal beliefs and values can sometimes lead to unintended stigma. This is especially problematic when the healthcare professional's values conflict with the patient's identity, sexual orientation, or health needs, such as when providing care for BBVs or STIs.

…from the experience of people that I work with, so, people living with hep B, so there is a fear from people living with hep B that the stigma will…that they will receive discrimination if they go to GPs in the same community. That fear actually translates into actions in a lot of people that they won't see the doctors in their community for hepatitis B. So, they will choose to go to another doctor, either like somewhere else further or go to hospital to be seen for hepatitis B, and their usual GP they only see them for other things, and they do not necessarily tell their usual GP about their hepatitis status. So, I cannot talk around whether that is correct from the side of the doctors, but the fear from people living with hep B does exist, and that happens a lot

(P19/female)

Perspectives shared by participants during the interviews further highlight how stereotypes held by healthcare providers about CALD people, and the resulting racialized stigma, constrain CALD people's engagement with healthcare. When healthcare providers approach individuals based on assumptions, prejudices, or generalised perceptions of their cultural or ethnic background, it can lead to stigma and discrimination, which in turn can hinder trust and engagement with care.

So, they [people of CALD backgrounds] have talked about being seen as not literate, not understanding about health care and be[ing] stigmatised, be[ing] stereotyped of not trusting doctor, not listening and not adhering to doctor's advice

(P4/female)

The experience or feeling of being treated with less dignity at the health service may be driven or perceived to be driven by language barrier. This is because people from CALD communities may be experiencing or stereotyped as having difficulties communicating in English. This barrier was evident across all our interview sessions and explored in the next theme.

### 3.4 Removing Language Barriers

Language is a well‐known barrier to care, making it difficult for people from CALD communities to engage with healthcare systems [12, 13, 14, 40]. However, the issue is not just about speaking a different language but also about how this barrier may be impacting trust, understanding, and the ability to advocate for one's own health. Language barriers may prevent individuals from expressing their symptoms clearly, understanding medical advice, or following through on treatment regimens [83].

One participant described language as “the first barrier” that needs to be addressed to ensure appropriate care for members of CALD communities. Common strategies used in health care to overcome language barriers involve translation and the use of interpreter services. However, the perspectives shared during the interviews suggest that these strategies are not very effective in helping people of CALD backgrounds to engage fully with healthcare. An important insight from the interviews is that while translation and interpretation could be useful to communicate information, it is doubtful if they can communicate meanings and feelings, even at their optimal use.

…because I speak Thai, Indonesian and Chinese, when there is a person from CALD background who comes and if they speak the same language, it's easier for us to communicate. In this society settings, it [English] is the first line of communication, but there's a lot of things that can't actually be translated through English. Like there's a lot of the nitty‐gritty and the feeling that's really hard to express, especially if you are a foreigner, I mean speak English as a second language. So, being able to connect and talk to them [people from CALD communities] in their first language, is quite important

(P21/male/BBV living experience)

The interview data also indicate that people of CALD backgrounds may not have confidence in the quality of interpretation thereby impacting their engagement with healthcare. Experiences shared by participants show that people of CALD backgrounds who have good English language proficiency and who have used interpretation services have noted experiencing low quality interpretation thereby further highlighting how language can be a barrier whereby people of CALD backgrounds do not feel welcome by healthcare settings. This raises some concerns about whether health information is being accurately and appropriately translated to CALD communities, particularly those who may be less proficient in English.

when people talk about not feeling welcome in healthcare setting… it's still around…not wanting to see doctors not speaking the language because they feel the distance, hard to navigate and for those who experience interpretation in like interpreting [in] healthcare, I think they averagely rate the quality of interpretation really low…doctors are doing better job now in using less jargon in their work and using plain language better. So those people could understand the doctors in English already, but then when it was translated by the interpreter, they thought that the interpreters did it wrongly. So, they think it's not welcoming, it's not enabling for people to seek care

(P4/female)

Even when interpretation is correctly done, there may also be concern relating to confidentiality on the part of the interpreter. This fear of confidentiality makes it difficult for people of CALD backgrounds to access available healthcare:

…if they are going to use an interpreter, how are they to know that this interpreter is confidential? The service user is always wary…not speaking the language and not trusting the interpreting service to be confidential…they shy away from accessing services, and sometimes they bring their own family in as an interpreter, and that in itself is a whole set of complications, because they don't want their niece or their nephew to know this is the illness they're going through, but they have to bring the person in. So, that in itself is another psychological distress. And so, “You know what? I'm not going to go. Why should I go? It's just too hard. It's way too difficult for me to deal with this”

(P11/gender not disclosed)

Participants also raised concerns about how information is communicated to people of different CALD backgrounds. Using medical jargons can deter CALD people from accessing health services. A participant from a CALD community, who is also a GP at a BBVs/STIs clinic described this situation:

the doctors in the hospital, they are specialists, they talk very fast and you could not understand them. Even I have difficulty understanding them sometimes and I'm already good at the English language, but the people who are not very good in English, they need the doctors to speak slower, which doesn't happen and the doctors, the specialists, they speak in a medical language that the normal person won't understand. It's okay if I'm a health professional, but the normal person, the patient won't understand and it's really difficult for these patients to navigate the health system

(P16/female)

Considering that it is not practicable to have healthcare professionals for all language and cultural groups of CALD communities, interpretation and translation services remain a vital aspect of healthcare services for people of CALD backgrounds. However, it is important to take steps to ensure that these translation services are accurately and appropriately translating health information to CALD clients and that confidentiality is assured.

### 3.5 Involving CALD Community Members as Healthcare Providers

Interview participants shared the common view that involving healthcare providers from CALD communities in providing care to CALD people would improve patient engagement. One notable reason for engaging CALD people in providing care and occupying leadership position is for them to act as the “voice” of CALD people, to *advocate* for them in an Australian health system that “ignores” CALD people:

I believe that the CALD community is minoritised and not just minoritised, but ignored, [laughs] sorry, it's a much stronger word, but I think it's being ignored by the Australian health system…CALD community doesn't have a place. A big part of it is because I think the CALD community doesn't have a voice…so there's no one advocating for us

(P7/female)

Having CALD people as providers is believed to remove, or at least, reduce the first barrier a CALD person encounters in a healthcare setting which is language‐related barriers as well as racism:

[If] there's a white man in front of you, I mean Anglo‐Saxon people, “Oh my God! I need to speak English.” You will think that, “Oh, I have to watch out for my grammar. What if they don't understand my accent; if my accent is too thick? How can I pronounce this? How can I pronounce that?” But if you see other fellas who also speak English as a second language, I think it can be quite helpful. I think me personally, I find it helpful if I can speak in my first language. So, sometimes if an Asian person come[s] in, usually I will be the one that face them, just to [create] that sort of [welcoming environment]…. I mean a face that you know reduces the first barrier

(P21/male/BBV living experience)

This strategy is believed to be effective since healthcare providers from CALD backgrounds, as insiders, could be more likely to understand the experiences of CALD population and how to support them. The overall argument is that a mainstream provider who does not have a first‐hand or lived experience of being a person of CALD background may not fully understand the experience of the CALD population using their mainstream lens.

I cannot imagine how could a mainstream person who has never had an experience of being a migrant understand the cultural issues, can deal with this community discrimination incident, because that lack of understanding, lack of lived experience, like mainstream can only face the problem when it happens, they cannot really think about anticipated problem. So, the mainstream skills and ideas and lived experience are sometimes so far away and it's not compatible with the community that is in need for the service

(P2/female)

However, it is also likely that having a provider from one's CALD or religious community may constrain engagement with care, especially for community‐stigmatised condition such as BBVs and STIs. This is because these conditions are attributed to failure to adhere to the socio‐cultural ethos of the community [57, 82] making client to feel they will be judged by a provider from their community and subsequently, be discriminated or even ostracised. This was vividly described by one of the study participants:

we have people from all, GPs from all sorts of life, and we have those who are Christian, Muslims, and we have heard from our Muslim brothers and sisters who wouldn't like to go to their regular GP, who happens also to be a Muslim, for any sexual health issues because when it comes to sexual health, it's a no‐go zone because of their religion. Yeah. That's what I meant by that. So, everyone needs to be informed about HIV. Everyone needs to be informed. Even the religious leaders because they are also stigmatising our community. We have a lot of community members who normally tell me, “I wouldn't want [my community members] to know… I wouldn't like my community, where I go to church or where I go to mosque to know about my HIV status because they are going to separate me from the rest of them”

(P5/female/BBV living experience)

This insight and the ones preceding them have implications for efforts at individual, community and healthcare system level for improving engagement with BBVs/STIs services for people from CALD communities as discussed below.

## 4 Discussion

Our study was designed to gain a deeper understanding of barriers constraining people from various CALD communities in Australia from engaging in BBVs and STIs preventive and treatment services and how these barriers could be addressed. Our results indicate that stakeholders believe a shift in cultural orientation of healthcare would better reflect the socio‐cultural and religious beliefs of CALD communities, thus improving patient engagement. Though people of CALD backgrounds in Australia are from diverse ethnicities and nationalities, as noted in the data, they are likely to share a more collectivist cultural orientation that is different from the individualistic orientation known to be predominant in Western societies [84, 85]. Stakeholders believe that people from various CALD communities in Australia, especially communities where collectivist orientation is predominant, may find it difficult to engage with the Australian healthcare system which is driven by a more individualist orientation. The Australian healthcare system focuses on Western biomedical notions of health and illness and predominantly applies these understandings to all patients, including people from CALD backgrounds [86] who may largely hold different socio‐cultural views and beliefs about health and illness. In terms of health messaging, people from CALD communities are more likely to have a higher level of community attachment [85]. As such, public health messaging to promote BBVs/STIs preventive and treatment practices may resonate better for these communities if such messages appeal to their responsibility to their families and/or other community members [87, 88].

Moreover, since health is a human rights issue that must be prioritised despite cultural and/or migration status [15], it is essential to make the Australian health system more inclusive by adopting cross‐cultural approaches that consider and accommodate diverse cultural understanding and needs of different CALD communities in addressing their health and illness concerns. This could be done through active and non‐tokenistic participation of these diverse CALD communities in planning, implementing, and evaluating interventions to reflect their cultural perspectives as such active participation could enhance health service access among these communities [89]. Also, collectivist culture‐based information dissemination model, such as storytelling, could also be effective in communicating health information and messages to CALD communities [90], instead of the current dominant use of individualist orientation that may not resonate with these communities.

The results also show how experiences of stigma impact CALD people's engagement with healthcare services in Australia. BBVs and STIs are highly stigmatised in many CALD communities partly because of the perceived link between behavioural practices negating traditional socio‐sexual norms and these infections [14, 54, 57, 67, 91]. The high level of community stigma around BBVs and STIs makes it difficult for members of CALD communities who are living with these infections to disclose their status and/or seek healthcare, partly because they may feel judged, excluded, or ostracised by significant others. Unfortunately, there is still stigma related to BBVs and STIs in healthcare settings where improved knowledge about these infections is expected to make the setting stigma‐free. Thus, stigma in healthcare settings also affect CALD people's engagement with both preventive and treatment services. The experience of BBVs/STIs‐related stigma in health service for members of CALD communities may be further compounded by racial discrimination, migration status, sexual orientation, gender and/or social status. This intersectional stigma experience may further reinforce low engagement among members of these communities. The effect of stigmatising BBVs and STIs at both community and healthcare levels is that people from various CALD communities may avoid seeking information and engaging in screening and treatment for these infections [14, 54, 57, 67, 91]. Consequently, concerted efforts to address stigma at both community and healthcare levels are critical if people from CALD communities are to engage with care. Improving health literacy among people from various CALD communities through appropriate and effective health education and promotion [92, 93], and improving cultural competence and partnership among healthcare workers [40, 90, 94] are key to addressing stigma and discrimination at community and healthcare settings respectively.

Particularly, addressing stigma and discrimination at the health service level requires cultural sensitivity training and continuous upskilling around cultural competence. Providers' understanding of diverse socio‐cultural perspectives in various CALD communities––and seeing these perspectives as resources rather than deficits––could make health services appealing and welcoming to people from CALD communities [40]. Culturally competent providers with clear understanding of how to navigate CALD people's concerns, would contribute immensely to making health services acceptable and accessible for CALD communities. A clear understanding and valuing of CALD people's socio‐cultural orientation and understanding of health and illness could help build trust between people from diverse CALD communities and providers thereby making the health services more inclusive [90, 94, 95]. This inclusivity could improve CALD people's health outcomes, acceptance, satisfaction and greater engagement with healthcare.

Building a culturally competent health workforce requires significant partnerships and collaborations with CALD communities to ensure that cultural training does not reinforce or encourage stereotypes [40, 96, 97]. Also, effective collaborations with CALD people in building a culturally competent workforce could foster trust [98] and patient engagement [99]. Similarly, building trust and culturally competent workforce would also require engaging CALD people as providers and leaders in the health system, and ensuring the availability of accurate and appropriate translation services. These would further establish trust for CALD people that the health system is culturally safe and responsive to their health needs. Of note, in line with previous studies among other vulnerable populations [100, 101], our results showed that people from CALD communities may be reluctant seeking BBVs/STIs treatment and care from providers who come from their communities because of anticipated stigma.

This reluctance has implications for competency training and reflexivity for providers from CALD communities to not reinforce community‐level stigma at the healthcare setting. This way, people from CALD communities may have an added advantage of engaging with BBVs/STIs services from providers who share similar cultural understandings of health and illness and possibly language. Given that health providers who are from CALD backgrounds may also face experiences of intersectional stigma, including racism, future research should seek to include the perspectives and experiences of these health providers. Aside from efforts to address stigma, remove language barriers and adopt cross‐cultural approaches, it is critically important to make the Australian healthcare system easier for people of CALD communities to navigate. As our results suggested and in support of evidence found in other research [102], adopting a less formalised and institutionalised BBVs/STIs services could improve access and engagement. Also, removing structural barriers related to migration status and other *determinants* of social determinants of health could help improve the extent to which people from diverse CALD communities engage with BBVs/STIs health services in Australia.

## 5 Limitations

The results of this study need to be understood within some caveats. First, recruitment was largely done using researchers' and participants' professional networks consisting of people who advocate, support, and work with CALD communities. Future research should seek to interview people from CALD communities themselves to explore experiences of health care and of intersectional stigma. Additionally, due to time and resource constraints on the study, all interviews were conducted in English. Results may therefore not reflect the views of people who are less proficient in English. Importantly, one of the major concerns regarding the use of the term—“CALD” in Australian health‐related research is the tendency to homogenise a range of varying perspectives and experiences. This concern relates to our study, even though we made efforts to recruit participants across different CALD communities in all the Australian states and territories. Notably, we recruited participants from sub‐Saharan African communities who are known to be underrepresented in this area of scholarship [103]. However, our analysis and conclusion did not reflect specificity across the different CALD communities we recruited participants from due to concerns around protecting the confidentiality of participants. Despite these limitations, the results of this study provide exploratory insights to understand and guide further studies, policies, and interventions to make BBVs and STIs services acceptable, accessible, and sustainable for CALD people in Australia.

## 6 Conclusion

Our study aimed to explore barriers that constrain CALD people's engagement with BBV and STI health services in Australia and how these barriers might be addressed. Using a collaborative approach, we explored how to make health services more accessible and acceptable for this population. Our results underscore the need for the Australian health system to be more inclusive and flexible to meet the needs of diverse populations, by developing better understandings of different cultural perspectives around health care, addressing stigma and discrimination, and providing culturally appropriate and safe care. Our results also have implications for easing navigation of the health system especially for minoritised communities and population groups.

## Author Contributions

Interviews were conducted by DJ, KV, KOW, SO, and SY. SO wrote the first draft of the manuscript, with contributions from EC, LB, and RH. All authors contributed to the final version of the manuscript.

## Ethics Statement

Ethics approval for this study was granted by the University of New South Wales Human Research Ethics Committee (HC220669).

## Conflicts of Interest

The authors declare no conflicts of interest.

## Data Availability

Data analysed in this study may be available based on reasonable request and subject to ethics approval.
